# Potential DNA binding and nuclease functions of ComEC domains characterized *in silico*

**DOI:** 10.1002/prot.25088

**Published:** 2016-07-01

**Authors:** James A. Baker, Felix Simkovic, Helen M.C. Taylor, Daniel J. Rigden

**Affiliations:** ^1^ Department of BiochemistryInstitute of Integrative Biology, University of LiverpoolLiverpoolL69 7ZBUnited Kingdom; ^2^ Present address: James A. Baker's current address is Manchester Institute of BiotechnologyThe University of Manchester131 Princess Street, ManchesterM1 7DNUnited Kingdom

**Keywords:** bacterial competence, ComEC, evolutionary covariance, domain structure, protein modeling

## Abstract

Bacterial competence, which can be natural or induced, allows the uptake of exogenous double stranded DNA (dsDNA) into a competent bacterium. This process is known as transformation. A multiprotein assembly binds and processes the dsDNA to import one strand and degrade another yet the underlying molecular mechanisms are relatively poorly understood. Here distant relationships of domains in Competence protein EC (ComEC) of *Bacillus subtilis* (Uniprot: P39695) were characterized. DNA‐protein interactions were investigated *in silico* by analyzing models for structural conservation, surface electrostatics and structure‐based DNA binding propensity; and by data‐driven macromolecular docking of DNA to models. Our findings suggest that the DUF4131 domain contains a cryptic DNA‐binding OB fold domain and that the β‐lactamase‐like domain is the hitherto cryptic competence nuclease. Proteins 2016; 84:1431–1442. © 2016 The Authors Proteins: Structure, Function, and Bioinformatics Published by Wiley Periodicals, Inc.

## INTRODUCTION

Natural transformation is the uptake of free environmental double stranded DNA (dsDNA) that has been secreted by other bacteria or is the product of cell lysis.^1^, ^2^ Bacterial competence is the physiological state in which DNA uptake is possible, and can be natural or induced. Transformation facilitates both inter‐ and intraspecies DNA transfer.^3^, ^4^ The benefits of bacterial DNA uptake by competence can result from either the acquisition and incorporation of exogenous genetic material into their genome or its use as a food resource of carbon, nitrogen, and phosphorus.^4^, ^5^, ^6^ It has also been suggested that environmental DNA from closely related species can act as templates for DNA repair.^4^

The composition of the competence system varies from species to species.^1^, ^5^ Considering that competence has been implicated in the acquisition of antibiotic resistance,^7^, ^8^, ^9^ and that genome plasticity of the pathogen *Helicobacter pylori* depends on its natural transformation,^10^ surprisingly little information is available about the molecular structures and mechanisms of the competence proteins.

**Figure 1:**
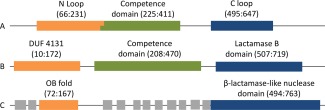
*B.subtilis* ComEC domain annotations. (A) Draskovic and Dubnau 12 define overlapping extracellular N Loop and the multitopic competence domains, followed by the extracellular C loop and an intracellular C terminal. (B) Pfam 11 domains (accessions PF13567, PF03772 and PF00753, respectively). (C) Approximate structure‐based domain definitions determined here along with transmembrane helices predicted by Phobius 83 (gray boxes; residue ranges 12–39, 45–64, 229–251, 263–283, 304–321, 327–345, 357–375, 387–409, 416–433, 445–467, 474‐492). [Color figure can be viewed in the online issue, which is available at wileyonlinelibrary.com.]

One *B. subtilis* protein, key for competence yet poorly understood, is ComEC. ComEC is composed of 776 residues and matches three domains in the Pfam database^11^ (Fig. 1); Domain of Unknown Function 4131 (DUF4131; PF13567) (residues 10–174), the competence domain (PF03772; residues 208–470), and the β‐lactamase‐like domain (PF00753; residues 507‐719). Previous characterization of ComEC revealed significant topological features^12^ and employed a different nomenclature (Fig. 1). Thus DUF4131 corresponds to the “N‐Loop”^12^ while the β‐lactamase‐like domain encodes for a “C‐loop” and a portion of the C‐terminal tail region (Fig. 1).^12^ The central competence domain was predicted to contain three transmembrane helices and a cytosolic amphipathic helix and is thought to be responsible for maintaining the competence membrane pore.^12^ Although dsDNA binds to the bacterial surface, only a single strand is taken up through the competence membrane pore while the other is degraded. In *Streptococcus pneumoniae,* 13 the EndA nuclease is known to carry out this degradation.^14^ However, the identity of the protein bearing this nuclease activity is not known in *B. subtilis* 15 or in other characterised species such as *T. thermophilus*.^16^ The competence domain has been shown to be essential for bacterial competence and is well conserved across competent species.^12^, ^17^, ^18^ It is proposed that ComEC exists as a homodimer held together by disulphide bonds, but other oligomeric structural arrangements cannot yet be ruled out.^12^ It is postulated that the competence domain maintains the pore structure.^12^ The function of the ComEC DUF4131 is unknown, yet it is essential for competence in *B. subtilis*: its deletion renders the bacterium incapable of transformation.^12^ Phylogenetic distributions^19^ suggest that ComEC (known as Rec2 in, for example, *Haemophilus influenzae* 20 and ComA in *Neisseria gonorrhoeae* 17) is one of the proteins most specifically associated with competence^14^ yet it has a distinct role in the human pathogen *Listeria* where it is required for the escape of infecting bacteria from host cell phagosomes.^21^

Here we probe ComEC structure and function *in silico* using a variety of homology, structural and covariance‐based bioinformatics methods. We can strongly assert that the β‐lactamase‐like domain in *B. subtilis* ComEC supports a nuclease function. We discover and describe a presumed single‐stranded DNA‐binding OB fold within DUF4131. The results enhance our understanding of bacterial competence machinery and will guide experimental structural biology efforts.

## MATERIALS AND METHODS

### Homology modeling

The webserver HHpred was used to search for distant homologues of ComEC domains^22^, ^23^ and to provide target‐template alignments for molecular modeling. Homology searches were done against the Protein Data Bank (PDB^24^) (server database version pdb70_06Sep14) using five iterations of HHblits^25^ (sequence database version uniprot20_2015_06) to generate the query Hidden Markov Model. The choice of templates, single or multiple, was driven by the quality of the models that resulted. For the β‐lactamase‐like domain, the structure of modular teichoic acid phosphorylcholine esterase (Pce; PDB code: 2bib^26^) was used as a single template. The ComEC β‐lactamase‐like domain model (ComEC residues M512‐N776) incorporated the two Zn^2+^ ions found in the template. Modeling of the OB fold of ComEC DUF4131 (V60‐H160) used two templates; subunit E of Replication Protein A (RPA14; PDB code 2pi2^27^), and subunit A of Human Replication Protein A (PDB code 3kdf, unpublished). PyMOL (http://www.pymol.org) was used to align and visualize protein structures. 500 models of the OB‐fold and 1000 models of the lactamase‐like domain were constructed using MODELLER 9.12^28^ and the five best according to DOPE^29^ were additionally analyzed by validation tools ProSA,^30^ Verify_3D^31^ (with a default sliding window averaging size of 21), and PROCHECK^32^ to select the best model.

### Covariance‐based domain decomposition and modeling

Using the ComEC DUF4131 sequence obtained from UniProt, evolutionary covariance analysis was used to predict residue‐residue contacts with PconsC2^33^ and a specialist β‐strand filtering protocol bbcontacts.^34^ PconsC2 is a meta‐predictor that takes 16 predictions as input: the PSICOV^35^ or plmDCA (Ekeberg *et al*. 2013, 2014) results from eight alignments, derived by Jackhmmer^36^ against the UniRef100 database or HHblits v2.0.15^25^ against the non‐redundant UniProt20 database v2013.03^37^ at *E*‐value cutoffs of 10^−40^, 10^−10^, 10^−4^, and 1. bbcontacts was applied to results from CCMpred,^38^ working with the results of an HHblits v2.0.15 database search of UniProt20 database v2013.03 with an *E*‐value cutoff of 10^−3^ and filtering to 90% sequence identity using HHfilter v2.0.15^25^ to reduce sequence redundancy. A joint PconsC2/bbcontacts contact prediction was derived by combining the two predictions and assigning a two‐fold higher weight to contacts predicted by both methods.^39^

The resulting contact maps informed the definition of two distinct domains within DUF4131. TMHMM^40^ was used to identify predicted transmembrane helices. Due to uncertainties in the domain boundaries, several different stretches were modelled. The predicted contacts were used to drive *ab initio* fragment‐based folding of regions mapping to the OB fold region in Rosetta^41^ using the PconsFold protocol.^42^ Modeling was performed with PconsC2 contact predictions alone or using the joint PconsC2/bbcontacts set. The PconsFold protocol employs the top‐L predicted contacts (where L is the target sequence length) to drive *ab initio* modelling. The resulting 1000 models were clustered using Spicker.^43^ The centroid model from the largest resulting cluster (which represents the most favored fold prediction) was searched for structural similarity to PDB entries using the DALI server.^44^ Centroid models were also subjected to Rosetta refinement using the default parameters of the relax command in Rosetta, after which the best scoring of the 20 resulting models was also searched against the PDB with DALI. Models are available from the authors on request.

### Model analysis

Electrostatic analysis was carried out using the APBS Tools plugin of PyMOL.^45^ Mapping of sequence conservation on to the final model was done using the ConSurf web server.^46^ Sequence conservation information was obtained from five iterations of PSI‐BLAST^47^ with an *E*‐value of 0.0001. The models, along with comparator crystal structures of known function were submitted to the structure‐based nucleic acid binding prediction servers DNABIND,^48^ and iDBPs.^49^

### DNA docking

B‐form double‐stranded (ds)DNA (default sequence CCCTGTGGAGCCACACCCTAG and its complementary strand) was generated at the make‐NA server.^50^ Data‐driven docking on the HADDOCK web server was carried out between the dsDNA and ComEC β‐lactamase‐like model using the default parameters.^51^ The server allows the specification of “active residues” presumed to be located near the protein‐ligand interface. H571 in the predicted catalytic site of the β‐lactamase‐like model was chosen. On the DNA side, active residues were specified halfway down the length of the DNA backbone to avoid docking to the termini. Passive residues, not involved in interface formation, were automatically selected by the server.^51^

## RESULTS

### DUF4131 contains an OB fold likely to bind DNA

**Figure 2:**
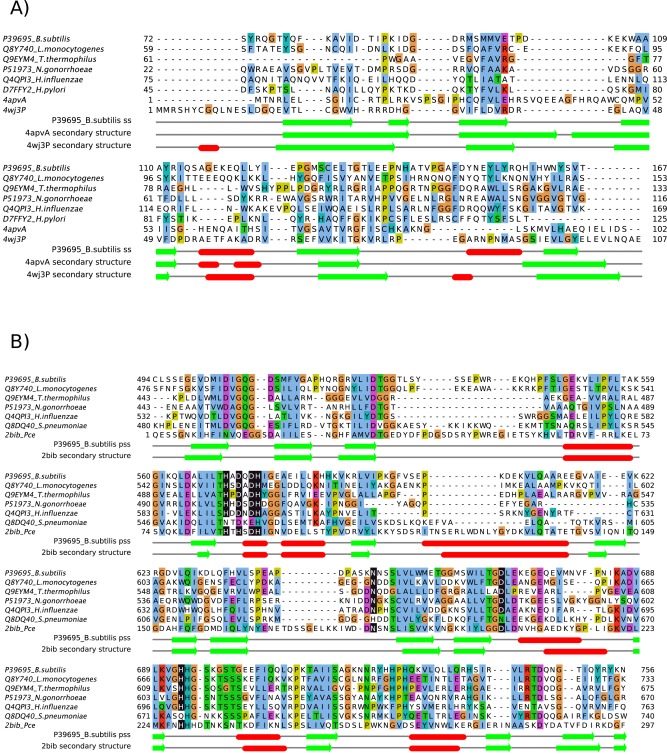
Alignments of (A) OB folds and (B) β‐lactamase‐like domains in ComEC and homologous proteins from species in which competence has been experimentally studied (above, labeled with UniProt 37 accession and species names) with structures (below, labelled with PDB codes). In (A) The *B. subtilis* ComEC OB fold model (Fig. 3b) was structurally aligned to the PDB entries using MUSTANG 84 and STACCATO 85. In (B) the alignment of ComEC with the template used for homology modelling derives from HHpred 23 and (predicted) Zn‐ligating residues are highlighted as white on black. Note that the corresponding positions in the *S. pneumoniae* ComEC sequence are not highlighted since it is unlikely that this sequence binds zinc (see text). Predicted secondary structure (pss; deriving from PSIPRED 86 run at the HHpred server) and actual secondary structure (assigned to the OB fold model and experimental structures by DSSP 87) are shown underneath the alignment as red bars (α‐helices) or green arrows (β‐strands). The figure was made with Jalview ^88^. [Color figure can be viewed in the online issue, which is available at wileyonlinelibrary.com.]

**Figure 3:**
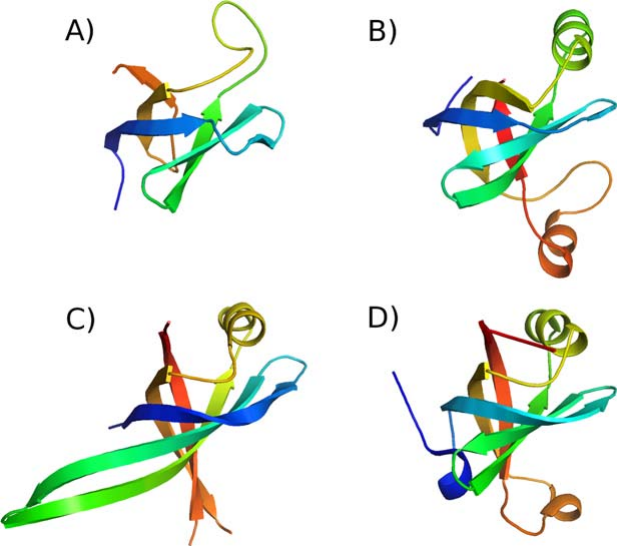
Models of the *B. subtilis* ComEC OB fold compared to crystal structures. Structures are shown coloured from blue (N‐terminus) to red (C‐terminus) (A) homology model of residues 72‐148. (B) covariance‐assisted fragment‐assembly model of residues 72‐167. (C) The crystal structure of Primosomal Replication Protein N *Klebsiella pneumonia* (PDB code 4apv; 56) D) the OB fold from subunit A of the structure of *Pseudomonas aeruginosa* Glutamyl‐tRNA(Gln) Amidotransferase (PDB code 4wj3; 58). [Color figure can be viewed in the online issue, which is available at wileyonlinelibrary.com.]

HHpred revealed distant homology between the *B. subtilis* ComEC DUF4131 sequence and proteins of the OB fold superfamily (Supporting Information Fig. 1a). The relationship was supported by a good match between the predicted secondary structure of the former and the actual secondary structure of the latter [Fig. 2(A)]. The ComEC sequence shared at most 15% sequence identity with these distant homologues but HHpred probabilities of up to 80% strongly support the fold assignment. For comparison, non‐OB folds scored probabilities of at best 34% indicating a clear distinction between the best matches and alternative putative fold matches. OB folds can support either single‐stranded (ss)DNA or oligosaccharide binding functions, but proteins that are known or suspected to bind to ssDNA were the best matches. Based on HHpred template rankings structures of human replication protein A 32 kDa subunit (PDB codes 2pi2 and 3kdf; Deng *et al*. 2007; unpublished) were used for model building. The final homology model of the DUF4131 OB fold [Fig. 3(A)] performed moderately well by protein validation measures. It achieved a *Z* score of −3.02 with ProSA^30^ which is within the range of values for experimental structures of this size (of around +1 to −8) and, by Verify_3D^31^, 81% of the residues scoring >0.2, indicative of good packing quality. A Ramachandran plot generated by PROCHECK^32^ placed 73% of residues in the most favored region with a single disallowed residue (Q122). This compares favorably with the 90% most favored expected of well‐refined crystal structures.^32^ The typical topology of the OB fold (Theobald *et al*. 2003) is largely found in the ComEC DUF4131 homology model [Fig. 3(A)] with the two three‐stranded antiparallel β‐sheets discernible. However, a helix often found between the third and fourth strand (top right in the orientation shown in Fig. 352^,^ 53) was not present.

Evolutionary covariance analysis was then employed to assess whether DUF4131 contained unsuspected multiple structural units^54^, ^55^—as also suggested by the HHpred results that matched only part of DUF4131 to OB folds—and to confirm the existence of the OB fold using homology‐independent fragment‐based modelling assisted by predicted contacts.^42^ Evolutionary covariance analysis was made possible by the large number of homologous sequences available for the ComEC DUF4131 region. For example, the 16 different alignments used by the metapredictor PconsC2 contained up to 11,621 sequences for the *B. subtilis* ComEC DUF4131 query (see also Supporting Information Table 1). Furthermore, there was a high degree of sequence diversity in the family resulting from its broad distribution across multiple bacterial phyla. For example, even after filtering to remove redundancy to a 90% sequence identity level, as done in the CCMpred/bbcontacts pipeline (Methods), 1057 sequences remain.

**Figure 4:**
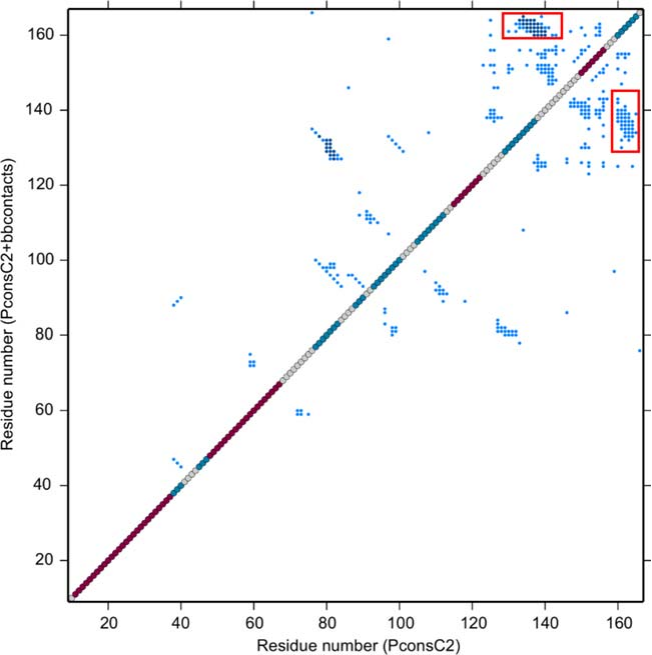
Joint contact maps resulting from combining contact predictions from PconsC2 alone (lower diagonal) or PconsC2 and bbcontacts (upper diagonal) for the B. *subtilis* ComEC DUF4131 sequence from residues 10–167. Red dots on the diagonal mark residues predicted to be α‐helical, while β‐strand residues are marked in mid‐blue. Off diagonal blue points represent predicted contacts between residues determined by evolutionary covariance analysis. In the upper panel, those in darker blue are those found in both the PconsC2 and bbcontacts lists. Predicted contacts suggesting an antiparallel pairing between β‐strands from residues 135–140 and residues 161–166 are boxed. [Color figure can be viewed in the online issue, which is available at wileyonlinelibrary.com.]

Predicted contact maps produced for the full Pfam definition of DUF4131 in ComEC (Fig. 4) immediately suggested the presence of two structural modules. The numerous predictions between pairs of residues in the range 70–172 are consistent with it representing a folded domain in which packing interactions are detected by the covariance analysis. The absence of any predicted contacts between 70–172 and 10–69, however, argues against the existence of any actual interactions between the two stretches in the native structure and so supports the notion of two independent structural units encompassed by a single Pfam DUF entry. Furthermore, the two helices predicted in the residue range 10–69 are both predicted to be membrane‐spanning by TMHMM,^40^ whereas the OB fold is a soluble, globular structural module.

Interestingly, while HHpred and covariance results agreed on the presence of a distinct two transmembrane helix unit at the N‐terminus of DUF4131, the predicted contact map suggested a different domain boundary at the C‐terminal limit of the OB fold than the distant homology inferred above from HHpred results. The OB fold in the homology model finished at around residue 148 whereas contact maps revealed that a predicted β‐strand from residues 135–140 paired in an antiparallel fashion with the predicted strand from residues 161–166 (Fig. 4). Each strand was strongly predicted by PSIPRED, at most one residue in each strand achieving a confidence rating of <8, with 9 being the maximum. Covariance‐assisted fragment‐based modeling was therefore carried out for several putative OB fold domain limits. The results consistently retrieved OB folds from the PDB with significant scores (Z‐scores >4;^44^) by structural similarity searches with DALI. The highest scoring DALI structural similarity matches were achieved when a residue range from 72–167 was modeled using a joint contact prediction from PconsC2 and bbcontacts and incorporating an additional refinement step in Rosetta (see Methods). The resulting model [Fig. 3(B)] picked out primosomal replication protein Prib from *Klebsiella pneumonia* [PDB code 4apv;^56^; Fig. 3(C)] as its nearest structural match with a *Z*‐score of 8.2. Other OB fold proteins with known ssDNA binding function also matched strongly with *Z*‐scores >7. Details are given in Supporting Information Figure 2, along with similar results for an alternative residue range tested. Unlike the earlier homology modeling, the covariance‐assisted fragment‐based modeling does not depend directly on fold information in the PDB, although it does assemble models from 3‐ and 9‐residue backbone fragments of experimentally determined structures. Generating fragment libraries using Rosetta's “exclude homologous fragments” option eliminated no structures as parents of fragments, confirming that the relationship between the ComEC OB fold and known structures is very distant. PDB structures within the SCOPe superfamily of OB‐fold nucleic acid‐binding proteins (b.40.4; 57), the group containing 4apv for example, were responsible for only around 0.5% of the fragments used in the modelling. Thus, since the modeling employed few fragments from nucleic acid‐binding OB folds, the emergence of these as the nearest DALI matches can be viewed as significant, and the covariance‐assisted fragment‐based modeling can be considered to provide strong, largely independent support for the assignment of an OB fold to this region of ComEC. The use of predicted contacts was not essential for the emergence of OB fold matches—DALI scores of up to 7.1 were obtained for unassisted models—but produced structures with a stronger resemblance to known OB folds.

These final covariance‐assisted models, such as that shown in Figure 3(B), demonstrated more regular packing of β‐strands into sheets than seen in the homology model [Fig. 3(A)], as well as revealing the characteristic helix between the third and fourth strands (top right in the orientation shown in Fig. 3). In the covariance‐assisted model, a further helix was present before last strand of the OB fold. This is not seen in the top DALI match [Fig. 3(C)], but is seen in the structure of the ligase domain of the asparagine transamidosome from *Pseudomonas aeruginosa* [PDB code 4wj3^58^; Fig. 3(D)] which matched the model with a DALI score of 6.6.

Independent evidence supporting nucleic acid binding function was sought using two protein structure‐based predictors of DNA binding, iDBPs^49^ and DNABIND.^48^ Results for the ComEC DUF4131 models, both homology‐ and covariance‐based were mixed. On the one hand, neither model achieves scores that would in themselves strongly indicate DNA binding. On the other, the model scores were better, by both methods, than those of known OB fold ssDNA binding comparator proteins tested. One possible explanation for these results is that dsDNA is likely to be the ligand for the majority of cases upon which predictive methods are trained. We are not aware of any methods specifically for prediction of ssDNA binding function.

**Figure 5:**
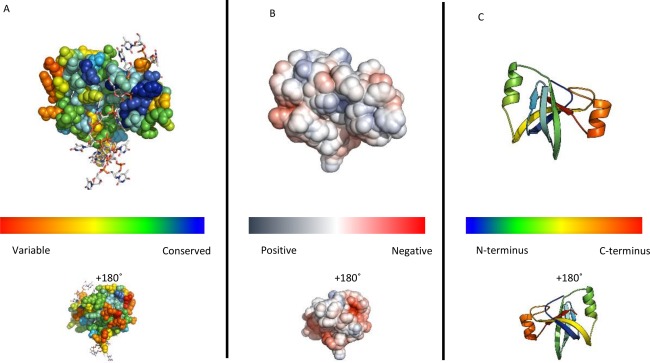
Representations of the ComEC OB fold. (A) Conservation scores from Consurf mapped onto a spacefill representation of the final contact‐assisted fragment‐based model of the ComEC OB fold coloured according to ConSurf 46 conservation scores from blue, most conserved, to red least. The ssDNA molecule from the structure of the *E. coli* PriB complex 59 is shown according to superposition of the two proteins. **(**B) The electrostatic surface potential of the ComEC OB fold using APBS electrostatic calculations at approximately ±5kT/e 45. (C) Cartoon representation of the model coloured from blue (N‐terminus) to red (C‐terminus). [Color figure can be viewed in the online issue, which is available at wileyonlinelibrary.com.]

Sequence conservation mapping onto the final contact‐assisted model structure revealed moderate conservation of a surface patch, but no strong positive charge on the face of the OB fold expected to bind nucleic acid (Fig. 5). The absence of stronger conservation may be related to the expected lack of specificity toward DNA sequence: maintenance of general DNA affinity may be consistent with greater sequence divergence than a sequence‐specific binding function. Overlaid ssDNA, as seen in the structure of *Escherichia coli* PriB (PDB code 2ccz;^59^), coincides with the protein model indicating that binding in a similar fashion would require a conformational change of the ComEC structure. This is highly plausible, however, since the domain excursion that would be required to reorganize—positioned on the right in Figure 5—is known to undergo conformational change on ssDNA binding in other OB fold proteins.^60^

### The β‐lactamase‐like domain is a predicted nuclease

HHpred unambiguously confirmed the presence of a β‐lactamase like fold in ComEC [Supporting Information Fig. 1(b)] with probabilities reaching 100% for the closest structural match teichoic acid phosphorylcholine esterase (Pce; PDB code: 2bib, Hermoso *et al*. 2005), despite a modest 22% shared sequence identity. Models were constructed using this esterase as the single template The final ComEC β‐lactamase‐like domain model, incorporating two Zn^2+^ ions, scored reasonably well with ProSA (*z* scores = −5.92) within the range of values for experimental structures of this size (of around −2 to −11). The Ramachandran plot generated by PROCHECK showed 89% of residues in the most favored regions and a single Ramachandran‐disallowed residue (D536). By VERIFY_3D, 67% of residues had scores > 0.2 with the predicted catalytic site generally higher scoring.

**Figure 6:**
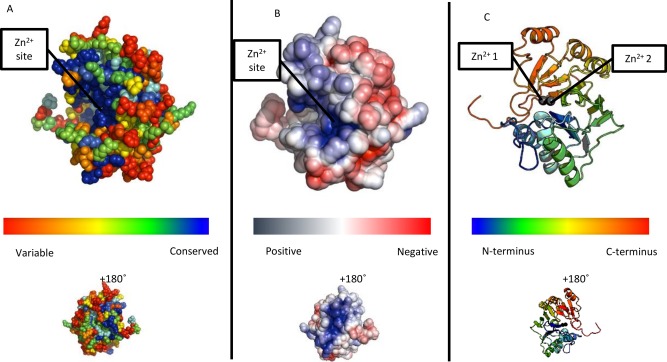
(A) Conservation scores from Consurf mapped onto a spacefill representation of β‐lactamase‐like domain of ComEC showing a region of highly conserved residues encompassing, but not limited to, the binding site for the two zinc ions 46. (B) The electrostatic surface potential of the ComEC β‐lactamase‐like domain using APBS electrostatic calculations at approximately ±5kT/e 45. Note the coincidence of the positively charged and conserved regions. (C) Cartoon representation of the model coloured from blue (N‐terminus) to red (C‐terminus). Grey spheres are the zinc ions.

Model analysis strongly suggested that the β‐lactamase‐like domain is catalytically active since Zn‐ligating residues present in the template structure are almost entirely conserved in ComEC sequences [Fig. 2(B)]. The interesting exception is His87 which is replaced by a conserved Asp in ComEC (Asp573 in *B. subtilis*). Asp residues are commonly found at Zn‐binding sites^61^ so that ComEC would be expected to maintain the same binuclear site as seen in the template. Maintenance of the ability to bind divalent cations suggests, since these metals are responsible for the key activation of a catalytic water molecule,^62^ that this domain in ComEC is catalytically active for hydrolysis. Outside the catalytic site conservation is seen more broadly when ComEC sequence variation is mapped onto the model structure [Fig. 6(A)], consistent with the existence of a larger conserved substrate binding site. This is significant since it is known that some members of the β‐lactamase‐like superfamily are catalytically inactive.^63^, ^64^ Well‐conserved residues outside the metal centers include positively charged Lys541, Lys690, His694, Arg722, His725, and Arg743.

**Figure 7:**
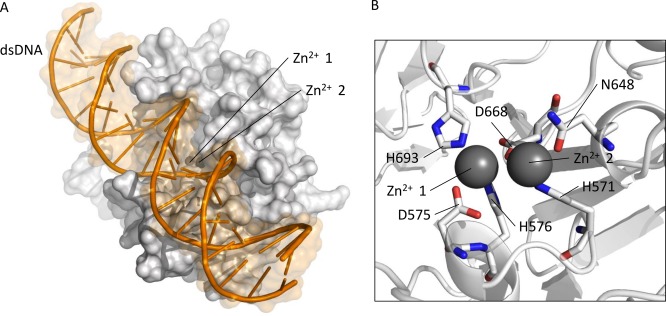
(A) B‐form dsDNA docked by data‐driven HADDOCK with the β‐lactamase‐like domain of ComEC shown in the same orientation as in Figure 5. The dsDNA is shown as a cartoon with a transparent orange surface. ComEC β‐lactamase‐like domain is shown as a white surface model. The Zn^2+^ ions are shown although they were not explicitly included in the docking. (B) Residues within a 4 Å distance of the Zn^2+^ ions, shown as space filling spheres, are shown as sticks with red and blue denoting oxygen and nitrogen respectively. [Color figure can be viewed in the online issue, which is available at wileyonlinelibrary.com.]

Given the functional context of ComEC in bacterial competence, and the existence of nucleic acid binding members of the β‐lactamase superfamily,^64^ evidence supporting DNA binding was again sought for the ComEC domain model. By iDBPs, the model scored 0.59 on a scale of 0–1 (1 being the highest DNA binding propensity), exceeding the scores assigned to known RNA cleaving enzymes with β‐lactamase‐like domains ‐ *Methanosarcina mazei* cleavage and polyadenylation specificity factor (CPSF; PDB code 2xr1^65^) with 0.53 and *B. subtilis* RNase Z (PDB code 1 year44^66^) with 0.50. These scores were superior to those of β‐lactamase‐like domains not active on nucleic acids such as the template Pce enzyme from *Streptococcus pneumoniae* with a score of 0.48. With DNABIND,^48^ similar results were seen, with known or suspected nucleic acid binding proteins (ComEC, CPSF, and RNase *Z* scoring 0.34, 1.42, and 0.16, respectively) clearly distinguishable from Pce with −1.67. In agreement with these predictions and known trends among DNA‐binding proteins,^48^, ^67^ examination of the electrostatic surface potential of the protein revealed that the conserved presumed catalytic site is strongly positively charged [Fig. 6(B)]. In some of these β‐lactamase superfamily members, the catalytic site is found at the interface between the main catalytic domain and an accessory domain such as the beta‐CASP domain in CPSF‐73.^68^ RNase Z from *B. subtilis* (PDB code 1 year44^66^) is a single domain β‐lactamase‐like nuclease, like ComEC, and is therefore a suitable comparison. The positive charge in ComEC [Fig. 6(B)] is more pronounced (not shown) than that seen for RNase Z. HADDOCK docking [Fig. 7(A)] revealed that dsDNA could be readily accommodated in the presumed catalytic site of the model [Fig. 7(B)]. The top cluster 1 (Fig. 7) contained 126 poses and had a HADDOCK score of −93.5 ± 3.6, corresponding to a *Z*‐Score of −1.9. Strikingly, in the dsDNA‐bound complex of the ComEC model, the Zn^2+^ ions are only 4.3 Å from the scissile phosphodiester bond.

Certain nucleases within the β‐lactamase superfamily can be recognised by their β‐CASP motifs.^69^ The β‐CASP region is now included in Pfam as a separate family (PF10996) and has been structurally characterized as a domain following the β‐lactamase‐like unit in these enzymes, with the catalytic site forming at the domain interface.^68^ There is no sign of this domain in ComEC: indeed, Pfam currently records no proteins bearing both β‐CASP and Competence (PF03772) domains. We therefore suggest that ComEC represents a distinct emergence of nuclease activity in the superfamily. Indeed, multiple families of β‐lactamase‐like enzymes bear nuclease activity.^70^

## DISCUSSION

We have described compelling evidence of an OB fold in the DUF4131 domain of ComEC. The evidence from distant homology was here supported by application of recently emerging covariance‐assisted modelling. The OB fold is a compact structural motif that was named originally due to its oligonucleotide or oligosaccharide binding properties.^52^ However, both distant homology detection with HHpred and structural similarity searches with covariance‐assisted models consistently matched ssDNA‐binding OB fold proteins most strongly. Thus, despite the equivocal results obtained with structure‐based DNA‐binding function predictors, it is very likely that it functions to bind ssDNA in ComEC, especially as this is in such obvious agreement with the broad functional context of ComEC. Some OB fold‐containing proteins such as telomer end‐binding proteins dimerise such that the OB folds act together to bind ssDNA,^71^ others bind as single domains to ssDNA. The fact that a dimer is a plausible oligomeric state for ComEC^12^ means that the former is a possibility, but distinguishing the two scenarios will require further research. Previous data have established that the DUF4131 domain (or the “N loop” in the terminology of Ref. 12) is extracellular. Taken together with the predicted ssDNA binding function assigned, this suggests that ssDNA is being handled extracellularly by ComEC. This is consistent with ComEC importing only a single strand of DNA into the cell, the other being degraded. By homology, OB folds are also predicted to be present in ComEC proteins and homologues from other species in which competence has been studied [Fig. 2(A)]. Curiously, however, the sequence from *Thermus thermophilus* appears to lack a region matching the first β‐strand and may, therefore, be more structurally and functionally divergent. We verified the absence in alignments from different software (not shown). Although DUF4131 domains most commonly precede Competence domains, as in ComEC (Fig. 3), Pfam records proteins with stand‐alone DUF4131 domains which would also, on the basis of our results, be predicted as DNA‐binding. The parsing of DUF4131 using evolutionary covariance analysis into two structural units, a pair of transmembrane helices and the OB fold, is a still‐unusual application of a long‐standing idea.^54^ Our unpublished data suggest that many other large DUFs cryptically harbor multiple structural modules.

The β‐lactamase‐like domain of ComEC is homologous to proteins in the β‐lactamase superfamily of protein domains that catalyze hydrolytic cleavage of various substrates. β‐lactamases are ancient proteins^72^ and are well known for their association with antibiotic resistance.^73^ The β‐lactamase superfamily comprises an exceptionally versatile group of proteins within which both bacterial and archaeal species have, multiple times independently, evolved the ability to hydrolytically cleave nucleic acids.^5^, ^65^, ^66^, ^74^ The model of the ComEC β‐lactamase‐like domain revealed a conserved putative catalytic site containing a full complement of ligands for maintenance of a binuclear zinc site. Furthermore, structure‐based DNA binding predictions and the context of ComEC as a DNA processing protein^1^, ^12^ strongly suggest that the β‐lactamase‐like domain in *B. subtilis* ComEC represents yet another independently evolved instance of nuclease activity in the β‐lactamase superfamily. The docking experiments with dsDNA showed striking structural complementarity between the model structure and ideal dsDNA resulting in a binding mode in which the scissile phosphodiester bond is placed only 4.3 Å from the catalytic Zn^2+^ ions, well‐positioned, given minor conformational changes, to undergo cleavage.

Although requiring experimental validation, this predicted nuclease domain appears to be a strong candidate for the currently unassigned activity in *B. subtilis* 15, 16 and other characterized species such as *T. thermophilus* 16 known to break down the alternate DNA strand as the other passes through the competence pore to the cytosol. ComEC homologues from other experimentally characterized species contain the predicted nuclease domain [Fig. 2(B)] with the curious exception of *Helicobacter pylori*. The shorter homologous sequence from *H. pylori* 75 terminates immediately before the predicted start of the nuclease domain and the genome contains no further proteins bearing any close relationship to *B. subtilis* ComEC β‐lactamase‐like domain. In *Streptococcus pneumoniae,* 13 the EndA nuclease is known to carry out the degradation of one strand as the other is passed to the cytosol.^14^ *S. pneumoniae* ComEC contains a full‐length β‐lactamase‐like domain but detailed examination shows nonconservative substitutions at many of the zinc‐binding positions [Fig. 2(B)]. At only two of the seven positions indicated in Figure 2(B) does the *S. pneumoniae* ComEC maintain the metal‐ligating positions, otherwise invariant across the ComEC proteins shown. At position 675, aligned with His693 in *B. subtilis* ComEC, for example, *S. pneumoniae* ComEC has a glutamine residue, an amino‐acid only very rarely found at zinc‐binding sites.^76^, ^77^ These changes would prevent zinc binding and hence render the domain inactive in the *S. pneumoniae* protein. Surprisingly, however, related Bacilli such as *Lactobacillus johnsonii* contain both a predicted orthologue (by reciprocal BLAST) of *S. pneumoniae* EndA and a ComEC protein which retains all the key metal‐binding residues. Adding a further layer of complexity, no EndA homologue is apparent in *H. pylori* suggesting that this bacterium, anomalous in other ways too,^14^ may employ even a third type of nuclease for this purpose.

The limited experimental characterization of ComEC largely owes to the work of Draskovic and Dubnau^12^ which addressed its topology, oligomerization and disulphide bonding. An intramolecular disulphide bond between residues 131 and 172 was demonstrated. The first of these residues lies within our final OB fold model [Fig. 3(B)], the latter lies later in the sequence but the relative positions of Cys 131 and the end of our model are consistent with disulphide bond formation. Under oxidizing conditions, inter‐molecular disulphide bonds were observed, with mutation of some of the other Cys residues affected in this process.^12^ Although various possibilities were considered a homodimeric structure was favored in which transmembrane helices from two subunits together formed a pore. A topological model was obtained using β‐galactosidase (LacZ) and alkaline phosphatase (PhoA) fusions with activity of each suggesting intracellular or extracellular localization of the fusion point, respectively. These data suggest an extracellular localization for the “N‐loop,” encompassing the OB fold (Fig. 1), in agreement with the role envisaged for this domain above. For the β‐lactamase‐like domain, however, part—the “C‐loop”—was predicted extracellular, but the latter part was predicted to be cytosolic. This domain can clearly not span the membrane, but the experimental data are considered more reliable for extracellular predictions^78^ suggesting that the catalytic domain lies outside the cell. Draskovic and Dubnau^12^ also compared their topology data with bioinformatic predictions of transmembrane helices. Current tools predict 11 transmembrane helices, rather than the seven noted at the time. With the exception of the last of the seven, all are still predicted, but five new helices are now annotated (Fig.1). Predictions have improved significantly in the past decade^79^ and the 11 can be considered a better estimate. However, the nine transmembrane helices predicted between the OB‐fold and β‐lactamase‐like domains, if true, are obviously inconsistent with their both being extracellular. Given the experimental data suggesting they are,^12^ including the existence of the intramolecular disulphide bridge, we suggest that the error lies with the transmembrane helix prediction. Suggestively, one helix (from residues 416–433) is much more weakly predicted than the others (Supporting Information Figure 3). Finally, Draskovic and Dubnau^12^ built on the knowledge that ComEC was essential for DNA uptake^80^ by assessing the importance of the “N‐loop,” containing the OB‐fold region identified here. They found that it was essential for transformation, confirming the functional importance of the domain, presumably for ssDNA handling.^12^

In summary, we provide important insights into ComEC domain structure and function predicting DNA binding and cleavage roles to the DUF4131 and β‐lactamase‐like domains, respectively, of *B. subtilis* ComEC. The key role of ComEC in competence has been known for 20 years,^81^ its role in the spread of antibiotic resistance is known^7^, ^8^, ^9^ and data show important roles for the protein in prominent human pathogens.^10^, ^21^ Nevertheless, relatively few experimental data have been obtained hitherto. By providing structural domain limits and testable functional hypotheses this work will significantly facilitate the future experimental characterization required to shed more light on this intriguing protein.

## Author Contributions

DJR conceived and supervised the study. HMCT and FS carried out the covariance analysis and the contact‐assisted fragment assembly modeling. JAB carried out all other experiments. All authors contributed to writing the manuscript.

## Supporting information

Supporting Information
