# Supplementary material for: Potential DNA binding and nuclease functions of ComEC domains characterized in silico
Source: Proteins. 2016 Jul 1;84(10):1431–42. doi: 10.1002/prot.25088 (PMC5031224; doi:10.1002/prot.25088)
Supplement: Supplementary file 1 — Supporting Information [file PROT-84-1431-s001.pdf]

## Supplementary Figures

**Fig 1. Raw HHpred server output for a) residues 1-191 containing the OB fold and b) residues 479-776, containing the β-lactamase-like nuclease domain, of *B. subtilis* ComEC.**

**a)**

| No | Hit | | Prob | E-value | P-value | Score | SS | Cols | Query HMM | Template HMM | |
|---|---|---|---|---|---|---|---|---|---|---|---|
| 1 | 3kdf_D | Replication protein A 3 | 79.5 | 14 | 0.00038 | 25.2 | 7.7 | 64 | 77-142 | 33-97 | (132) |
| 2 | 3au7_A | TIAS, putative uncharac | 77.8 | 11 | 0.0003 | 31.6 | 7.6 | 62 | 77-139 | 286-348 | (402) |
| 3 | 3kf6_A | Protein STN1; OB fold, | 71.0 | 29 | 0.0008 | 24.6 | 9.1 | 66 | 77-144 | 49-116 | (159) |
| 4 | 4gn4_B | Obody AM2EP06; beta bar | 68.5 | 26 | 0.00072 | 23.0 | 7.1 | 64 | 77-142 | 21-88 | (108) |
| 5 | 4gnx_B | Putative uncharacterize | 65.0 | 35 | 0.00096 | 23.2 | 7.5 | 65 | 77-142 | 38-102 | (136) |
| 6 | 2pi2_A | Replication protein A 3 | 60.9 | 47 | 0.0013 | 25.9 | 7.7 | 63 | 78-142 | 74-137 | (270) |
| 7 | 1c0a_A | Aspartyl tRNA synthetas | 58.8 | 58 | 0.0016 | 28.7 | 8.6 | 69 | 77-147 | 17-86 | (585) |
| 8 | 4wj4_A | Aspartate--tRNA(Asp/Asn | 55.2 | 53 | 0.0015 | 29.0 | 7.8 | 69 | 77-147 | 26-95 | (599) |
| 9 | 4joi_A | CST complex subunit STN | 52.9 | 65 | 0.0018 | 22.6 | 8.7 | 64 | 77-142 | 38-125 | (166) |
| 10 | 1sr3_A | APO-CCME; OB fold, beta | 52.9 | 66 | 0.0018 | 22.6 | 9.5 | 56 | 77-139 | 30-88 | (136) |
| 11 | 3m4p_A | Ehasnrs, asparaginyl-tR | 51.0 | 1.1E+02 | 0.003 | 25.9 | 8.8 | 72 | 77-149 | 32-109 | (456) |
| 12 | 3e0e_A | Replication protein A; | 49.2 | 53 | 0.0015 | 20.5 | 5.5 | 56 | 77-139 | 13-78 | (97) |
| 13 | 3nem_A | Aspartyl-tRNA synthetas | 45.5 | 1.5E+02 | 0.0043 | 24.8 | 9.4 | 68 | 77-146 | 18-87 | (438) |
| 14 | 4rmf_A | Aspartate--tRNA(Asp/Asn | 44.7 | 1.4E+02 | 0.0038 | 26.5 | 8.7 | 67 | 77-147 | 18-85 | (609) |
| 15 | 110w_A | Aspartyl-tRNA synthetas | 41.6 | 1.2E+02 | 0.0035 | 26.5 | 7.9 | 67 | 77-147 | 18-84 | (580) |
| 16 | 1e1o_A | Lysyl-tRNA synthetase, | 41.5 | 1.5E+02 | 0.0043 | 25.5 | 8.3 | 64 | 77-142 | 66-132 | (504) |
| 17 | 3kf8_A | Protein STN1; OB fold; | 37.7 | 1.4E+02 | 0.0038 | 22.6 | 6.8 | 62 | 77-138 | 87-158 | (220) |
| 18 | 1j6q_A | Cytochrome C maturation | 37.5 | 1.2E+02 | 0.0034 | 21.2 | 8.2 | 56 | 77-139 | 34-93 | (136) |
| 19 | 4gnx_A | Putative uncharacterize | 37.0 | 1.1E+02 | 0.003 | 20.5 | 8.5 | 65 | 77-154 | 20-96 | (114) |
| 20 | 2xgt_A | Asparaginyl-tRNA synthe | 36.1 | 1.4E+02 | 0.0039 | 25.1 | 7.2 | 71 | 77-149 | 13-84 | (435) |
| 21 | 1eov_A | ASPRS, aspartyl-tRNA sy | 34.8 | 2.5E+02 | 0.0069 | 24.0 | 9.2 | 65 | 77-143 | 38-108 | (487) |
| 22 | 3ut1_A | Lethal(3)malignant brai | 33.5 | 37 | 0.001 | 27.5 | 3.1 | 23 | 137-159 | 209-231 | (324) |
| 23 | 1gut_A | Mopii, molybdate bindin | 33.0 | 87 | 0.0024 | 18.2 | 6.6 | 52 | 78-134 | 7-58 | (68) |
| 24 | 2kct_A | Cytochrome C-type bioge | 30.6 | 1.3E+02 | 0.0036 | 19.6 | 9.4 | 54 | 77-137 | 10-69 | (94) |
| 25 | 1wyd_A | Hypothetical aspartyl-t | 29.7 | 2.8E+02 | 0.0078 | 23.2 | 8.0 | 64 | 77-143 | 18-81 | (429) |
| 26 | 3a74_A | Lysyl-tRNA synthetase; | 29.3 | 1.9E+02 | 0.0053 | 24.8 | 7.0 | 64 | 77-142 | 58-123 | (493) |
| 27 | 2k50_A | Replication factor A re | 28.8 | 1.4E+02 | 0.0039 | 19.4 | 6.0 | 56 | 77-137 | 18-84 | (115) |
| 28 | 4ex5_A | Lysine--tRNA ligase; st | 27.2 | 1.8E+02 | 0.005 | 25.2 | 6.5 | 61 | 77-139 | 88-150 | (529) |
| 29 | 3i7f_A | Aspartyl-tRNA synthetas | 25.9 | 3.8E+02 | 0.01 | 23.3 | 8.9 | 67 | 77-145 | 64-133 | (548) |
| 30 | 1x54_A | Asparaginyl-tRNA synthe | 25.7 | 2.7E+02 | 0.0074 | 23.3 | 7.2 | 64 | 77-142 | 18-83 | (434) |
| 31 | 1fr3_A | MOP, molybdate/tungstat | 23.9 | 1.3E+02 | 0.0037 | 17.4 | 6.3 | 53 | 78-136 | 7-59 | (67) |
| 32 | 4j15_A | Aspartate--tRNA ligase, | 23.1 | 4.2E+02 | 0.012 | 22.9 | 9.0 | 68 | 77-147 | 79-149 | (521) |

b)

| | | | | | | | | | | | |
|---|---|---|---|---|---|---|---|---|---|---|---|
| 1 | 2bib_A | CBPE, teichoic acid pho | 100.0 | 4.2E-33 | 1.2E-37 | 264.4 | 28.0 | 245 | 16-281 | 1-297 | (547) |
| 2 | 3bv6_A | Metal-dependent hydrola | 99.9 | 4.7E-25 | 1.3E-29 | 200.0 | 18.3 | 241 | 17-298 | 58-372 | (379) |
| 3 | 2gcu_A | Putative hydroxyacylglu | 99.9 | 1E-26 | 2.8E-31 | 198.6 | 6.6 | 216 | 19-272 | 1-244 | (245) |
| 4 | 2wyl_A | L-ascorbate-6-phosphate | 99.9 | 5.1E-25 | 1.4E-29 | 198.4 | 17.7 | 248 | 17-298 | 34-347 | (360) |
| 5 | 1xm8_A | Glyoxalase II; structur | 99.9 | 9.7E-25 | 2.7E-29 | 187.3 | 11.5 | 196 | 21-266 | 1-209 | (254) |
| 6 | 3adr_A | Putative uncharacterize | 99.9 | 2.1E-25 | 6E-30 | 191.8 | 6.1 | 214 | 19-268 | 2-258 | (261) |
| 7 | 1qh5_A | Glyoxalase II, protein | 99.9 | 4.6E-23 | 1.3E-27 | 177.5 | 15.7 | 170 | 21-233 | 1-189 | (260) |
| 8 | 4ysb_A | Metallo-beta-lactamase | 99.9 | 2.4E-24 | 6.7E-29 | 182.6 | 7.6 | 201 | 19-262 | 1-222 | (233) |
| 9 | 2qed_A | Hydroxyacylglutathione | 99.9 | 4E-23 | 1.1E-27 | 177.7 | 13.3 | 196 | 19-259 | 6-237 | (258) |
| 10 | 4chl_A | Persulfide dioxygenase | 99.9 | 3.7E-24 | 1E-28 | 182.0 | 5.6 | 210 | 13-262 | 1-230 | (238) |
| 11 | 2p18_A | Glyoxalase II; metallop | 99.9 | 8.5E-22 | 2.4E-26 | 173.9 | 17.3 | 174 | 21-233 | 29-242 | (311) |
| 12 | 1zkp_A | Hypothetical protein BA | 99.9 | 3.2E-21 | 8.9E-26 | 166.5 | 19.7 | 216 | 17-273 | 21-266 | (268) |
| 13 | 3md7_A | Beta-lactamase-like; ss | 99.9 | 3.7E-22 | 1E-26 | 174.7 | 12.6 | 218 | 15-283 | 23-291 | (293) |
| 14 | 2xf4_A | Hydroxyacylglutathione | 99.9 | 9.3E-22 | 2.6E-26 | 163.6 | 13.5 | 177 | 21-225 | 1-201 | (210) |
| 15 | 1k07_A | FEZ-1 beta-lactamase; m | 99.8 | 2.4E-20 | 6.6E-25 | 160.4 | 16.1 | 177 | 23-233 | 17-232 | (263) |
| 16 | 3g1p_A | Protein PHNP; C-P lyase | 99.8 | 3E-20 | 8.2E-25 | 159.7 | 16.4 | 208 | 19-283 | 1-251 | (258) |
| 17 | 3kl7_A | Putative metal-dependen | 99.8 | 1.2E-19 | 3.4E-24 | 153.7 | 17.1 | 199 | 15-269 | 24-235 | (235) |
| 18 | 1sml_A | Protein (penicillinase) | 99.8 | 1.8E-19 | 4.9E-24 | 155.4 | 18.3 | 165 | 23-220 | 30-230 | (269) |
| 19 | 4jo0_A | CMLA; nonheme oxygenase | 99.8 | 2.6E-20 | 7.3E-25 | 174.9 | 13.3 | 232 | 18-274 | 250-534 | (534) |
| 20 | 2cbn_A | Ribonuclease Z; phospho | 99.8 | 1.6E-19 | 4.4E-24 | 158.7 | 17.5 | 212 | 21-273 | 2-304 | (306) |
| 21 | 1y44_A | Ribonuclease Z; zinc-de | 99.8 | 2.8E-19 | 7.8E-24 | 158.3 | 18.0 | 213 | 21-273 | 1-302 | (320) |
| 22 | 4ax1_B | Metallo-beta-lactamase | 99.8 | 1.7E-19 | 4.7E-24 | 158.2 | 16.4 | 210 | 2-258 | 6-279 | (303) |
| 23 | 2vw8_A | PA1000, PQSE; quinolone | 99.8 | 6.8E-22 | 1.9E-26 | 173.6 | 1.0 | 229 | 10-263 | 1-278 | (303) |
| 24 | 2zwr_A | Metallo-beta-lactamase | 99.8 | 8.4E-20 | 2.3E-24 | 151.5 | 13.4 | 170 | 21-225 | 1-193 | (207) |
| 25 | 3m8t_A | 'BLR6230 protein; subcl | 99.8 | 2.9E-19 | 8.1E-24 | 156.2 | 15.8 | 171 | 23-227 | 47-253 | (294) |
| 26 | 3vpe_A | Metallo-beta-lactamase; | 99.8 | 8.7E-19 | 2.4E-23 | 150.5 | 16.0 | 194 | 23-258 | 18-251 | (262) |
| 27 | 5aeb_A | LRA-12; hydrolase, carb | 99.8 | 3.2E-19 | 8.8E-24 | 154.2 | 13.3 | 169 | 23-225 | 30-236 | (272) |
| 28 | 3tp9_A | Beta-lactamase and rhod | 99.8 | 1.9E-19 | 5.4E-24 | 167.8 | 12.0 | 182 | 18-232 | 1-225 | (474) |
| 29 | 2i7t_A | Cleavage and polyadenyl | 99.8 | 1.8E-18 | 5.1E-23 | 160.6 | 16.4 | 216 | 18-265 | 9-270 | (459) |
| 30 | 4efz_A | Metallo-beta-lactamase | 99.8 | 1.6E-18 | 4.5E-23 | 152.0 | 14.4 | 175 | 18-218 | 2-218 | (298) |
| 31 | 2e7y_A | TRNAse Z; tRNA maturati | 99.8 | 2.9E-18 | 7.9E-23 | 148.9 | 15.5 | 211 | 21-273 | 1-278 | (280) |
| 32 | 3zq4_A | Ribonuclease J 1, RNAse | 99.8 | 2.1E-17 | 5.9E-22 | 156.7 | 20.7 | 212 | 16-267 | 4-256 | (555) |
| 33 | 4ysl_A | Beta-lactamase domain p | 99.8 | 4.1E-18 | 1.1E-22 | 149.2 | 14.7 | 170 | 24-219 | 11-215 | (294) |
| 34 | 2ohh_A | Type A flavoprotein FPR | 99.8 | 2.2E-18 | 6E-23 | 157.1 | 13.0 | 207 | 30-263 | 34-288 | (404) |
| 35 | 1e5d_A | Rubredoxin\:oxygen oxid | 99.8 | 1.1E-18 | 3.1E-23 | 159.1 | 10.4 | 201 | 31-263 | 35-284 | (402) |
| 36 | 2zo4_A | Metallo-beta-lactamase | 99.8 | 3E-17 | 8.3E-22 | 145.0 | 19.0 | 182 | 23-230 | 8-247 | (317) |
| 37 | 3r2u_A | Metallo-beta-lactamase | 99.8 | 2.8E-18 | 7.8E-23 | 159.6 | 12.9 | 180 | 14-232 | 20-245 | (466) |
| 38 | 3jxp_A | Coenzyme PQQ synthesis | 99.8 | 4.4E-18 | 1.2E-22 | 150.7 | 13.3 | 220 | 21-273 | 3-303 | (321) |
| 39 | 3iek_A | Ribonuclease TTHA0252; | 99.8 | 5.1E-18 | 1.4E-22 | 156.4 | 13.9 | 214 | 21-265 | 1-249 | (431) |
| 40 | 3bk2_A | RNAse J, metal dependen | 99.8 | 3.7E-17 | 1E-21 | 155.2 | 20.1 | 211 | 15-267 | 11-272 | (562) |
| 41 | 1ycg_A | Nitric oxide reductase; | 99.8 | 3.5E-18 | 9.6E-23 | 155.6 | 12.5 | 202 | 31-263 | 36-283 | (398) |
| 42 | 4ojv_A | 3',5'-cyclic-nucleotide | 99.8 | 5E-18 | 1.4E-22 | 153.0 | 12.1 | 217 | 21-273 | 4-367 | (369) |
| 43 | 4ad9_A | Lactb2, beta-lactamase- | 99.7 | 2E-17 | 5.4E-22 | 144.4 | 13.4 | 171 | 28-229 | 29-213 | (289) |
| 44 | 4qn9_A | N-acyl-phosphatidyletha | 99.7 | 2.6E-16 | 7.2E-21 | 143.0 | 18.8 | 230 | 18-273 | 122-384 | (393) |
| 45 | 4c1d_A | Beta-lactamase class B | 99.7 | 9.9E-17 | 2.7E-21 | 136.2 | 14.0 | 167 | 31-236 | 45-241 | (242) |
| 46 | 5a0t_A | Ribonuclease J; hydrola | 99.7 | 8.3E-16 | 2.3E-20 | 146.0 | 21.8 | 209 | 18-267 | 16-266 | (561) |
| 47 | 2az4_A | Hypothetical protein EF | 99.7 | 1.8E-16 | 5E-21 | 146.0 | 16.7 | 210 | 16-266 | 2-282 | (429) |
| 48 | 4eyb_A | Beta-lactamase NDM-1; m | 99.7 | 2.6E-17 | 7.3E-22 | 142.0 | 10.0 | 181 | 2-219 | 15-253 | (270) |
| 49 | 2i7x_A | Protein CFT2; polyadeny | 99.7 | 4E-17 | 1.1E-21 | 158.6 | 11.8 | 215 | 21-277 | 1-249 | (717) |
| 50 | 1vjn_A | Zn-dependent hydrolase | 99.7 | 6.2E-17 | 1.7E-21 | 135.7 | 11.3 | 173 | 18-258 | 10-195 | (220) |

**Fig 2. Raw DALI scores for a) the chosen final model, encompassing residues 72-167 of *B. subtilis* ComEC (see Fig 3B) and b) the top cluster centroid model for residues 72-172. Each is the top cluster centroid resulting from contact-assisted (PconsC2/bbcontacts predictions) *ab initio* Rosetta modelling.**

**a)**

| No: | Chain | Z | rmsd | lali | nres | %id | PDB | Description |
|---|---|---|---|---|---|---|---|---|
| 1: | 4apv-A | 8.2 | 2.4 | 76 | 97 | 8 | PDB | MOLECULE: PRIMOSOMAL REPLICATION PROTEIN N; |
| 2: | 1woc-B | 8.1 | 2.5 | 79 | 99 | 6 | PDB | MOLECULE: PRIMOSOMAL REPLICATION PROTEIN N; |
| 3: | 1woc-D | 7.9 | 2.4 | 78 | 99 | 6 | PDB | MOLECULE: PRIMOSOMAL REPLICATION PROTEIN N; |
| 4: | 3afq-A | 7.8 | 2.5 | 77 | 105 | 12 | PDB | MOLECULE: SINGLE-STRANDED DNA-BINDING PROTEIN; |
| 5: | 1z9f-A | 7.8 | 2.1 | 75 | 89 | 7 | PDB | MOLECULE: SINGLE-STRAND BINDING PROTEIN; |
| 6: | 1woc-A | 7.8 | 2.6 | 79 | 99 | 6 | PDB | MOLECULE: PRIMOSOMAL REPLICATION PROTEIN N; |
| 7: | 1x3f-B | 7.8 | 2.5 | 78 | 115 | 10 | PDB | MOLECULE: SINGLE-STRAND BINDING PROTEIN; |
| 8: | 3f2c-A | 7.7 | 2.2 | 75 | 993 | 7 | PDB | MOLECULE: GEOBACILLUS KAUSTOPHILUS DNA POLC; |
| 9: | 1x3e-B | 7.7 | 2.6 | 79 | 119 | 11 | PDB | MOLECULE: SINGLE-STRAND BINDING PROTEIN; |
| 10: | 2pnh-B | 7.7 | 2.3 | 73 | 94 | 7 | PDB | MOLECULE: PRIMOSOMAL REPLICATION PROTEIN N; |
| 11: | 4ycw-B | 7.7 | 2.6 | 75 | 502 | 8 | PDB | MOLECULE: LYSINE--TRNA LIGASE; |
| 12: | 4ycw-A | 7.7 | 2.8 | 76 | 502 | 8 | PDB | MOLECULE: LYSINE--TRNA LIGASE; |
| 13: | 3f2b-A | 7.6 | 2.2 | 75 | 994 | 7 | PDB | MOLECULE: DNA-DIRECTED DNA POLYMERASE III ALPHA CHAIN; |
| 14: | 1ue7-C | 7.6 | 2.4 | 76 | 98 | 11 | PDB | MOLECULE: SINGLE-STRAND BINDING PROTEIN; |
| 15: | 3afq-C | 7.6 | 2.2 | 75 | 105 | 11 | PDB | MOLECULE: SINGLE-STRANDED DNA-BINDING PROTEIN; |
| 16: | 3f2d-A | 7.6 | 2.2 | 75 | 993 | 7 | PDB | MOLECULE: 5'-D(*DCP*DAP*DGP*DTP*DGP*DAP*DGP*DAP*DCP*DGP*DGP |
| 17: | 1woc-C | 7.6 | 2.7 | 79 | 100 | 6 | PDB | MOLECULE: PRIMOSOMAL REPLICATION PROTEIN N; |
| 18: | 2pnh-A | 7.6 | 2.3 | 75 | 96 | 7 | PDB | MOLECULE: PRIMOSOMAL REPLICATION PROTEIN N; |
| 19: | 2dud-B | 7.6 | 2.5 | 78 | 92 | 8 | PDB | MOLECULE: SINGLE-STRANDED DNA-BINDING PROTEIN; |
| 20: | 2ccz-B | 7.6 | 2.7 | 79 | 118 | 8 | PDB | MOLECULE: 5'-D(*TP*TP*TP*TP*TP*TP*TP*TP*TP*TP*TP |
| 21: | 1txy-B | 7.6 | 2.1 | 70 | 84 | 7 | PDB | MOLECULE: PRIMOSOMAL REPLICATION PROTEIN N; |
| 22: | 3afq-B | 7.5 | 2.6 | 77 | 113 | 10 | PDB | MOLECULE: SINGLE-STRANDED DNA-BINDING PROTEIN; |
| 23: | 3eiv-A | 7.5 | 2.2 | 74 | 98 | 12 | PDB | MOLECULE: SINGLE-STRANDED DNA-BINDING PROTEIN 2; |
| 24: | 3afq-D | 7.5 | 2.3 | 75 | 107 | 12 | PDB | MOLECULE: SINGLE-STRANDED DNA-BINDING PROTEIN; |
| 25: | 3koj-A | 7.5 | 2.2 | 73 | 89 | 11 | PDB | MOLECULE: UNCHARACTERIZED PROTEIN YCF41; |
| 26: | 4dam-D | 7.4 | 2.9 | 80 | 109 | 10 | PDB | MOLECULE: SINGLE-STRANDED DNA-BINDING PROTEIN 1; |
| 27: | 4dam-J | 7.4 | 3.0 | 81 | 108 | 10 | PDB | MOLECULE: SINGLE-STRANDED DNA-BINDING PROTEIN 1; |
| 28: | 1ue6-C | 7.4 | 2.5 | 79 | 106 | 10 | PDB | MOLECULE: SINGLE-STRAND BINDING PROTEIN; |
| 29: | 1ue6-D | 7.4 | 2.5 | 79 | 104 | 10 | PDB | MOLECULE: SINGLE-STRAND BINDING PROTEIN; |
| 30: | 2dud-A | 7.4 | 2.5 | 78 | 95 | 8 | PDB | MOLECULE: SINGLE-STRANDED DNA-BINDING PROTEIN; |
| 31: | 1v1q-B | 7.4 | 2.6 | 79 | 112 | 8 | PDB | MOLECULE: PRIMOSOMAL REPLICATION PROTEIN N; |
| 32: | 2vw9-A | 7.3 | 2.3 | 78 | 108 | 10 | PDB | MOLECULE: SINGLE-STRANDED DNA BINDING PROTEIN; |
| 33: | 2vw9-B | 7.3 | 2.3 | 76 | 105 | 11 | PDB | MOLECULE: SINGLE-STRANDED DNA BINDING PROTEIN; |
| 34: | 3vdy-B | 7.3 | 2.2 | 72 | 97 | 7 | PDB | MOLECULE: SINGLE-STRANDED DNA-BINDING PROTEIN SSBB; |
| 35: | 1ue1-B | 7.3 | 2.7 | 79 | 119 | 10 | PDB | MOLECULE: SINGLE-STRAND BINDING PROTEIN; |
| 36: | 3vdy-A | 7.3 | 2.5 | 77 | 101 | 6 | PDB | MOLECULE: SINGLE-STRANDED DNA-BINDING PROTEIN SSBB; |
| 37: | 4dam-E | 7.2 | 2.9 | 80 | 112 | 10 | PDB | MOLECULE: SINGLE-STRANDED DNA-BINDING PROTEIN 1; |
| 38: | 3k80-D | 7.2 | 2.1 | 75 | 101 | 8 | PDB | MOLECULE: ANTIBODY; |
| 39: | 3eiv-D | 7.2 | 2.2 | 73 | 100 | 11 | PDB | MOLECULE: SINGLE-STRANDED DNA-BINDING PROTEIN 2; |
| 40: | 4dam-H | 7.2 | 2.6 | 78 | 108 | 9 | PDB | MOLECULE: SINGLE-STRANDED DNA-BINDING PROTEIN 1; |
| 41: | 2ccz-A | 7.2 | 2.5 | 77 | 121 | 8 | PDB | MOLECULE: 5'-D(*TP*TP*TP*TP*TP*TP*TP*TP*TP*TP*TP |
| 42: | 1x3g-B | 7.2 | 2.6 | 78 | 115 | 12 | PDB | MOLECULE: SINGLE-STRAND BINDING PROTEIN; |
| 43: | 1s3o-B | 7.2 | 2.4 | 78 | 104 | 6 | PDB | MOLECULE: SINGLE-STRANDED DNA-BINDING PROTEIN, |
| 44: | 1ue5-B | 7.2 | 2.4 | 75 | 109 | 9 | PDB | MOLECULE: SINGLE-STRAND BINDING PROTEIN; |
| 45: | 1txy-A | 7.2 | 2.0 | 71 | 92 | 7 | PDB | MOLECULE: PRIMOSOMAL REPLICATION PROTEIN N; |
| 46: | 3a5u-B | 7.1 | 2.5 | 79 | 112 | 11 | PDB | MOLECULE: SINGLE-STRANDED DNA-BINDING PROTEIN; |
| 47: | 3tqy-D | 7.1 | 2.5 | 77 | 98 | 10 | PDB | MOLECULE: SINGLE-STRANDED DNA-BINDING PROTEIN; |
| 48: | 1ue7-D | 7.1 | 2.2 | 73 | 95 | 10 | PDB | MOLECULE: SINGLE-STRAND BINDING PROTEIN; |
| 49: | 3klw-B | 7.1 | 2.4 | 74 | 90 | 9 | PDB | MOLECULE: PRIMOSOMAL REPLICATION PROTEIN N; |
| 50: | 3koj-B | 7.1 | 1.8 | 69 | 90 | 12 | PDB | MOLECULE: UNCHARACTERIZED PROTEIN YCF41; |

**b)**

| No: | Chain | Z | rmsd | lali | nres | %id | PDB | Description |
|---|---|---|---|---|---|---|---|---|
| 1: | 3dm4-B | 4.7 | 2.8 | 69 | 90 | 7 | PDB | MOLECULE: PRIMOSOMAL REPLICATION PROTEIN N; |
| 2: | 3klw-B | 4.7 | 2.8 | 69 | 90 | 7 | PDB | MOLECULE: PRIMOSOMAL REPLICATION PROTEIN N; |
| 3: | 2hql-B | 4.5 | 2.4 | 70 | 101 | 9 | PDB | MOLECULE: HYPOTHETICAL PROTEIN MG376 HOMOLOG; |
| 4: | 2hql-E | 4.4 | 2.7 | 69 | 98 | 4 | PDB | MOLECULE: HYPOTHETICAL PROTEIN MG376 HOMOLOG; |
| 5: | 2hql-D | 4.3 | 2.3 | 69 | 104 | 4 | PDB | MOLECULE: HYPOTHETICAL PROTEIN MG376 HOMOLOG; |
| 6: | 2hql-C | 4.3 | 2.5 | 65 | 91 | 5 | PDB | MOLECULE: HYPOTHETICAL PROTEIN MG376 HOMOLOG; |
| 7: | 3pgz-A | 4.3 | 3.1 | 73 | 109 | 7 | PDB | MOLECULE: SINGLE-STRANDED DNA-BINDING PROTEIN; |
| 8: | 2dud-B | 4.3 | 2.8 | 69 | 92 | 10 | PDB | MOLECULE: SINGLE-STRANDED DNA-BINDING PROTEIN; |
| 9: | 1sru-C | 4.3 | 3.0 | 71 | 96 | 10 | PDB | MOLECULE: SINGLE-STRAND BINDING PROTEIN; |
| 10: | 3koj-B | 4.2 | 3.2 | 69 | 90 | 6 | PDB | MOLECULE: UNCHARACTERIZED PROTEIN YCF41; |
| 11: | 2hql-A | 4.2 | 2.3 | 69 | 104 | 7 | PDB | MOLECULE: HYPOTHETICAL PROTEIN MG376 HOMOLOG; |
| 12: | 3dm4-A | 4.2 | 3.1 | 69 | 98 | 7 | PDB | MOLECULE: PRIMOSOMAL REPLICATION PROTEIN N; |
| 13: | 2dud-A | 4.2 | 2.9 | 72 | 95 | 10 | PDB | MOLECULE: SINGLE-STRANDED DNA-BINDING PROTEIN; |
| 14: | 3k8a-B | 4.1 | 3.1 | 70 | 103 | 14 | PDB | MOLECULE: PUTATIVE PRIMOSOMAL REPLICATION PROTEIN; |
| 15: | 3k8a-A | 4.1 | 3.2 | 70 | 100 | 14 | PDB | MOLECULE: PUTATIVE PRIMOSOMAL REPLICATION PROTEIN; |
| 16: | 3fhw-A | 4.1 | 3.2 | 69 | 100 | 7 | PDB | MOLECULE: PRIMOSOMAL REPLICATION PROTEIN N; |
| 17: | 3k81-D | 4.1 | 3.1 | 72 | 106 | 13 | PDB | MOLECULE: MP18 RNA EDITING COMPLEX PROTEIN; |
| 18: | 3klw-A | 4.1 | 3.1 | 69 | 98 | 7 | PDB | MOLECULE: PRIMOSOMAL REPLICATION PROTEIN N; |
| 19: | 3fhw-B | 4.1 | 3.2 | 69 | 100 | 7 | PDB | MOLECULE: PRIMOSOMAL REPLICATION PROTEIN N; |
| 20: | 1s3o-B | 4.1 | 3.1 | 72 | 104 | 10 | PDB | MOLECULE: SINGLE-STRANDED DNA-BINDING PROTEIN, |
| 21: | 3lgj-A | 4.1 | 2.7 | 66 | 95 | 9 | PDB | MOLECULE: SINGLE-STRANDED DNA-BINDING PROTEIN; |
| 22: | 3pgz-B | 4.0 | 2.8 | 69 | 114 | 7 | PDB | MOLECULE: SINGLE-STRANDED DNA-BINDING PROTEIN; |
| 23: | 2vw9-B | 4.0 | 3.0 | 69 | 105 | 13 | PDB | MOLECULE: SINGLE-STRANDED DNA BINDING PROTEIN; |
| 24: | 1woc-A | 4.0 | 3.7 | 71 | 99 | 7 | PDB | MOLECULE: PRIMOSOMAL REPLICATION PROTEIN N; |
| 25: | 1woc-D | 4.0 | 3.3 | 71 | 99 | 6 | PDB | MOLECULE: PRIMOSOMAL REPLICATION PROTEIN N; |
| 26: | 1woc-B | 4.0 | 3.8 | 71 | 99 | 7 | PDB | MOLECULE: PRIMOSOMAL REPLICATION PROTEIN N; |
| 27: | 1s3o-A | 4.0 | 2.9 | 71 | 107 | 10 | PDB | MOLECULE: SINGLE-STRANDED DNA-BINDING PROTEIN, |
| 28: | 2ccz-B | 4.0 | 3.5 | 74 | 118 | 5 | PDB | MOLECULE: 5'-D(*TP*TP*TP*TP*TP*TP*TP*TP*TP*TP |
| 29: | 3ull-B | 4.0 | 3.1 | 72 | 103 | 8 | PDB | MOLECULE: DNA BINDING PROTEIN; |
| 30: | 1wyd-A | 4.0 | 3.0 | 68 | 428 | 1 | PDB | MOLECULE: HYPOTHETICAL ASPARTYL-TRNA SYNTHETASE; |
| 31: | 2vl6-C | 4.0 | 2.8 | 70 | 259 | 10 | PDB | MOLECULE: MINICHROMOSOME MAINTENANCE PROTEIN MCM; |
| 32: | 3vdy-B | 4.0 | 3.2 | 69 | 97 | 13 | PDB | MOLECULE: SINGLE-STRANDED DNA-BINDING PROTEIN SSBB; |
| 33: | 3koj-A | 4.0 | 3.4 | 68 | 89 | 6 | PDB | MOLECULE: UNCHARACTERIZED PROTEIN YCF41; |
| 34: | 3jc7-5 | 4.0 | 2.7 | 71 | 654 | 4 | PDB | MOLECULE: DNA REPLICATION LICENSING FACTOR MCM2; |
| 35: | 1sru-B | 4.0 | 3.3 | 70 | 97 | 11 | PDB | MOLECULE: SINGLE-STRAND BINDING PROTEIN; |
| 36: | 3nel-A | 3.9 | 3.0 | 68 | 439 | 3 | PDB | MOLECULE: ASPARTYL-TRNA SYNTHETASE; |
| 37: | 4fdb-A | 3.9 | 3.2 | 67 | 95 | 12 | PDB | MOLECULE: PROBABLE PRIMOSOMAL REPLICATION PROTEIN N; |
| 38: | 3ulp-C | 3.9 | 3.1 | 73 | 116 | 8 | PDB | MOLECULE: SINGLE-STRAND BINDING PROTEIN; |
| 39: | 3ulp-A | 3.9 | 3.1 | 73 | 114 | 8 | PDB | MOLECULE: SINGLE-STRAND BINDING PROTEIN; |
| 40: | 3ulp-D | 3.9 | 3.1 | 73 | 113 | 8 | PDB | MOLECULE: SINGLE-STRAND BINDING PROTEIN; |
| 41: | 3k80-C | 3.9 | 2.9 | 71 | 103 | 13 | PDB | MOLECULE: ANTIBODY; |
| 42: | 3nem-A | 3.9 | 3.0 | 68 | 439 | 3 | PDB | MOLECULE: ASPARTYL-TRNA SYNTHETASE; |
| 43: | 3ulp-B | 3.9 | 3.1 | 73 | 114 | 8 | PDB | MOLECULE: SINGLE-STRAND BINDING PROTEIN; |
| 44: | 3stb-C | 3.9 | 3.0 | 71 | 111 | 10 | PDB | MOLECULE: SINGLE DOMAIN ANTIBODY VHH; |
| 45: | 3jc6-5 | 3.9 | 3.0 | 70 | 254 | 3 | PDB | MOLECULE: DNA REPLICATION LICENSING FACTOR MCM2; |
| 46: | 1wyd-B | 3.9 | 3.0 | 68 | 429 | 1 | PDB | MOLECULE: HYPOTHETICAL ASPARTYL-TRNA SYNTHETASE; |
| 47: | 1v1q-B | 3.9 | 3.3 | 74 | 112 | 5 | PDB | MOLECULE: PRIMOSOMAL REPLICATION PROTEIN N; |
| 48: | 2vl6-A | 3.9 | 2.8 | 70 | 259 | 10 | PDB | MOLECULE: MINICHROMOSOME MAINTENANCE PROTEIN MCM; |
| 49: | 3ull-A | 3.9 | 2.9 | 71 | 106 | 10 | PDB | MOLECULE: DNA BINDING PROTEIN; |
| 50: | 2vl6-B | 3.9 | 2.8 | 70 | 259 | 10 | PDB | MOLECULE: MINICHROMOSOME MAINTENANCE PROTEIN MCM; |

**Fig.3 Phobius transmembrane helix prediction for *B. subtilis* ComEC**

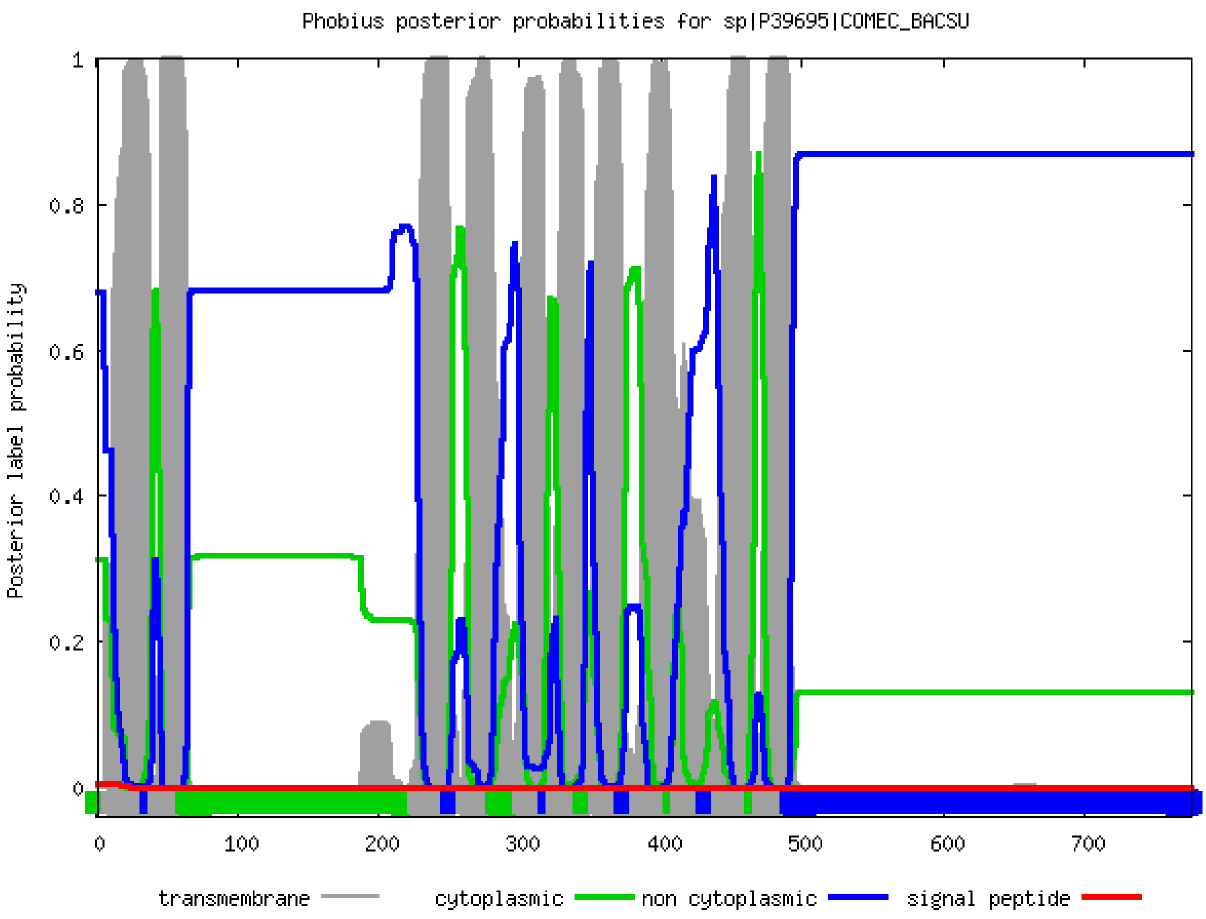

**Supplementary Table 1:** Numbers of sequences in alignments used by the metapredictor PconsC2 to predict contacts for DUF4131 region of *B. subtilis* ComEC. Figures exclude the query sequence.

| E-value | Number of sequences in alignment (HHblits) | Number of sequences in alignment (Jackhmmer) |
|---|---|---|
| 1 | 4478 | 11621 |
| 1e-4 | 2180 | 11039 |
| 1e-10 | 1262 | 3028 |
| 1e-40 | 0 | 93 |
